# The Effects of Hypocapnia on Brain Tissue Pulsations

**DOI:** 10.3390/brainsci10090614

**Published:** 2020-09-06

**Authors:** Meshal Alharbi, Poppy Turner, Jonathan Ince, Mitsuhiro Oura, Kelechi U. Ebirim, Alanoud Almudayni, Andrea Lecchini-Visintini, Jatinder S. Minhas, Emma M.L. Chung

**Affiliations:** 1 Cerebral Haemodynamics in Ageing and Stroke Medicine (CHiASM) Research Group, Department of Cardiovascular Sciences, University of Leicester, Leicester LE2 7LX, UK; meoa1@leicester.ac.uk (M.A.); pt178@leicester.ac.uk (P.T.); ji46@student.le.ac.uk (J.I.); aa1171@leicester.ac.uk (A.A.); jm591@leicester.ac.uk (J.S.M.); 2 College of Applied Medical Sciences, King Saud bin Abdulaziz University for Health Sciences, Riyadh 14611, Saudi Arabia; 3 School of Engineering, University of Leicester, Leicester LE1 7RH, UK; kue2@leicester.ac.uk (K.U.E.); alv1@leicester.ac.uk (A.L.-V.); 4 Nihon Kohden Corporation, Tokorozawa-shi, Saitama 359-0037, Japan; mitsuhiro_oura@mb1.nkc.co.jp; 5 College of Applied Medical Sciences, King Sattam bin Abdulaziz University for Health Sciences, Kharj 11564, Saudi Arabia; 6 University Hospitals of Leicester NHS Trust, Leicester LE1 5WW, UK; 7 NIHR Leicester Biomedical Research Centre, Leicester LE5 4PW, UK

**Keywords:** hypocapnia, brain tissue pulsations, BTP, cerebral autoregulation

## Abstract

Hypocapnia is known to affect patients with acute stroke and plays a key role in governing cerebral autoregulation. However, the impact of hypocapnia on brain tissue pulsations (BTPs) is relatively unexplored. As BTPs are hypothesised to result from cerebrovascular resistance to the inflow of pulsatile arterial blood, it has also been hypothesised that cerebral autoregulation changes mediated by hypocapnia will alter BTP amplitude. This healthy volunteer study reports measurements of BTPs obtained using transcranial tissue Doppler (TCTD). Thirty participants underwent hyperventilation to induce mild hypocapnia. BTP amplitude, EtCO_2_, blood pressure, and heart rate were then analysed to explore the impact of hypocapnia on BTP amplitude. Significant changes in BTP amplitude were noted during recovery from hypocapnia, but not during the hyperventilation manoeuvre itself. However, a significant increase in heart rate and pulse pressure and decrease in mean arterial pressure were also observed to accompany hypocapnia, which may have confounded our findings. Whilst further investigation is required, the results of this study provide a starting point for better understanding of the effects of carbon dioxide levels on BTPs. Further research in this area is needed to identify the major physiological drivers of BTPs and quantify their interactions with other aspects of cerebral haemodynamics.

## 1. Introduction

Carbon dioxide (CO_2_) levels affect vascular smooth muscle tone and hence play a pivotal role in cerebral autoregulation [1,2,3]. However, the impact of CO_2_ on brain tissue pulsations (BTPs) is poorly understood. Cerebral autoregulation is a homeostatic mechanism, which maintains cerebral blood flow through regulation of various body systems, including the cardiovascular, respiratory, and nervous systems [4]. A prominent mechanism underlying cerebral autoregulation control is blood vessel vasoconstriction and vasodilation, which provide a mechanism for controlling cerebral blood flow (CBF). It is well documented that increased arterial pressure of carbon dioxide (PaCO_2_) is associated with vasodilation [2]. Studies measuring cerebral blood flow using transcranial Doppler (TCD) previously found a sigmoidal association between hypercapnia (high PaCO_2_) and increased cerebral blood flow velocity [2].

BTPs are of increasing research and clinical interest. Over the last few decades, BTPs have been separately investigated using magnetic resonance imaging (MRI) and ultrasound methods [5]. Studies have found that BTP changes occur in various clinical conditions (such as orthostatic hypotension, depression, and in the presence of white matter hyperintensity) [6,7,8,9]. However, few studies have investigated the effects of natural physiological changes on brain tissue pulsations [5]. This can result in difficulties in data interpretation for evaluating the clinical applicability and reproducibility of BTP measurement methods.

Unlike TCD measurement of cerebral blood flow, transcranial tissue Doppler (TCTD) aims to measure the movement of brain tissue. This could be advantageous for clinical monitoring, as TCD relies on the detection of relatively weak ultrasound backscatter generated by red blood cells moving through the major arteries [10]. Consequently, adequate TCD measurements of blood flow can only be acquired through specific thin portions of the skull (e.g., the temporal bone window) and are unobtainable in approximately 1 in 10 subjects. Using this alternative TCTD measurement technique, since ultrasound backscatter from tissue is greater than from blood, different regions of the brain can be visualised through any position on the skull [11].

It has been theorised that BTP generation can be described by a plethysmographic-like model, where pulsatile cerebral arterial blood supply exceeds venous drainage in systole, causing tissue expansion and relaxation over the cardiac cycle [11]. This results in pulsing of the brain with every heartbeat. As this pulsing of the brain relies mainly on cerebral blood flow, it is likely that physiological changes affecting cerebral haemodynamics could also impact BTPs.

A previous study by Kucewicz et al. investigated the relationship between hypocapnia and BTPs in four healthy subjects who were asked to hyperventilate for 20 min [10]. Brain tissue pulsatility was found to decrease with hyperventilation; a fall in EtCO_2_ from ~40 mmHg to ~20 mmHg resulted in BTP reductions ranging between −25% and −50% [10]. The aim of this study was to further investigate the impact of brief periods of hyperventilation-induced mild hypocapnia (aimed at reducing PaCO_2_ by ~5 mmHg) on BTPs in a larger cohort of 30 healthy volunteers.

As PaCO_2_ varies considerably between individuals, and in cerebrovascular and non-cerebrovascular disease states [12], it is important to explore the impact of CO_2_ on BTPs in a larger cohort. This will facilitate physiological modelling of BTPs and will be used to assess whether there is a need to adjust BTP measurements for confounding by PaCO_2_. The results of this study will also be used to guide future BTP analysis in both healthy and diseased individuals.

## 2. Materials and Methods

### 2.1. Subjects and Measurements

Healthy participants were recruited from university staff, students, and their relatives, in line with a protocol approved by the University of Leicester Medicine and Biological Sciences Ethics Committee (Reference: 18110). Recruitment exclusion criteria included participants under 18 years old, and those with a significant past medical history of cardiovascular, respiratory, or neurological illness. Written informed consent was obtained from all participants.

**Figure 1:**
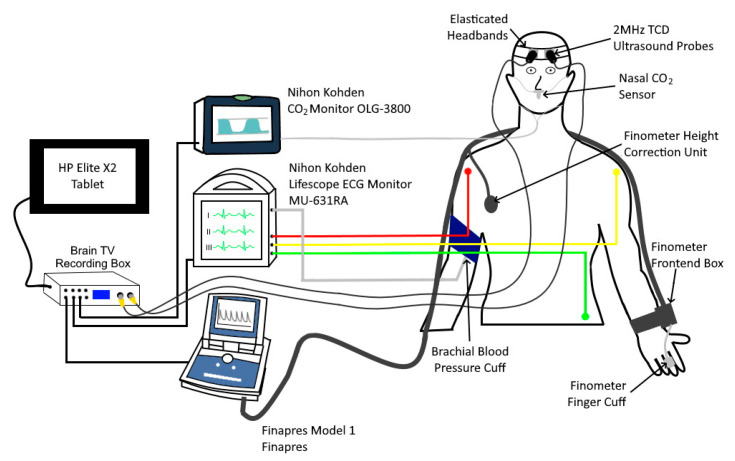
A schematic diagram of the experimental set-up.

Measurements were collected in a distraction-free laboratory, maintained at a target ambient temperature of 24 °C. BTPs were measured using a Brain Tissue Velocimetry (Brain TV) TCTD prototype (Nihon Kohden, Japan). This system is designed for bilateral monitoring and is equipped with a pair of standard 2 MHz TCD probes, held in place on the forehead using a bespoke headset designed ‘in house’. Probes were placed symmetrically on the forehead approximately 1 cm above the orbit. As with conventional TCD measurement of blood flow, both left and right sides were monitored. TCTD data are recorded alongside additional ECG, blood pressure (BP), and CO_2_ measurements from external monitoring equipment. Physiological measurements were obtained using a Nihon Kohden Lifescope Monitor to acquire three-lead electrocardiogram (ECG) and brachial blood pressure data. A Nihon Kohden OLG-3800 capnograph was used to record EtCO_2_ (a surrogate of PaCO_2_), and a Finapres Model 1 was used to obtain continuous blood pressure measurements. All signals were sampled at a frequency of 160 Hz and recorded for further analysis using ‘in house’ data analysis software and a Graphical User Interface (GUI) developed in MATLAB (version 2018b, The MathWorks Inc.). Figure 1 illustrates the equipment setup.

### 2.2. Experimental Protocol

The experimental protocol was adapted from a previous study involving TCD measurement of middle cerebral artery (MCA) cerebral blood flow to explore cerebral haemodynamic responses to CO_2_ levels [2]. An initial 1-min baseline recording was taken at rest with participants lying supine at 30°. This recording was then used to determine each participant’s respiratory rate at rest and a metronome was set to increase their resting respiratory rate by five breaths per minute. Participants were asked to hyperventilate for 90 s, breathing in time with the metronome, with the aim of inducing a target EtCO_2_ reduction of 5 mmHg. Hyperventilation was followed by a 2-min recovery period. A second hyperventilation period aiming to achieve an EtCO_2_ reduction of 10 mmHg (by further increasing the respiratory rate) was attempted. However, this was difficult to implement due to participant protocol adherence difficulties.

### 2.3. Data Analysis

The in-phase and quadrature-phase (IQ) data from each TCTD recording were down-sampled from 8 kHz to 160 Hz to reduce the size of our files, giving a temporal resolution of 6.25 ms. Tissue velocity at each depth was estimated using an autocorrelator [13] and integrated over time to obtain a BTP signal representing real-time tissue displacement at each tissue depth. The BTP signals were then filtered to remove respiration using a high pass filter with cut-off at 75% of the mean cardiac cycle frequency. Heart rate was estimated by calculating the mean cardiac frequency using each subject’s ECG R-wave interval.

BTP signals were displayed in MATLAB with the R-R wave intervals overlaid and traces manually inspected to allow the user to remove any cardiac cycles containing artefacts. Artefacts were defined as any noticeable perturbations not regularly repeating with the cardiac cycle. Similarly to artefacts in TCD measurements of cerebral blood flow, other artefacts result from motion of the probe or of the participant during recordings. Further analysis was based on these cleaned (artefact free) data.

Inspection of the quality of the recordings showed that good quality signals were obtained through the forehead for the first 20 gates, corresponding to depths within the brain ranging from 22 to 60 mm. Signal quality weakened beyond this depth, therefore only data from the first 20 gates were analysed. A small number of additional gates (one to three of the deepest gates) were removed during analysis of recordings from 5/30 participants due to limited penetration of the ultrasound beam in specific subjects.

For each recording, a Bulk BTP signal was calculated by averaging signals across all available depths. The Bulk BTP signal shows collective displacement of brain tissue in the direction of the beam over time (corresponding to the instantaneous bulk movement of tissue located at a depth of 20–60 mm from the face of the probe). A further quantity describing the magnitude of each pulsation, beat-to-beat Bulk BTP amplitude, was estimated by calculating the absolute difference between the peak and the trough of the Bulk BTP signal for each cardiac cycle.

The remaining physiological measurements (BP, ECG, and EtCO_2_) were analysed to provide beat-to-beat estimates of pulse pressure (PP), mean arterial pressure (MAP), heart rate (HR) and EtCO_2_. HR was calculated by measuring the length of the subject’s R-R interval and determining the frequency per minute, which gives an instantaneous HR for each cardiac cycle.

### 2.4. Statistical Analysis

**Figure 2:**
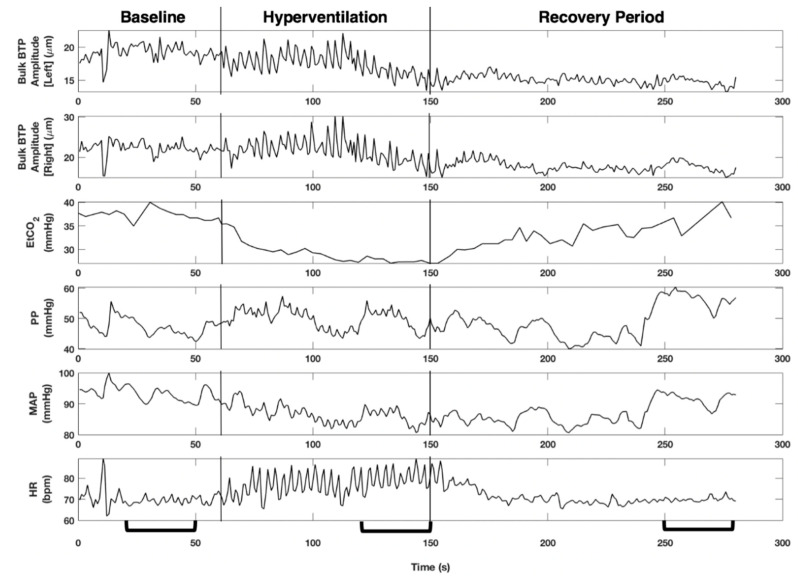
Time series for each variable are shown for an entire recording in which a 26-year-old male performs the hyperventilation manoeuvre. A reduction in EtCO_2_ and an increase in heart rate (HR) can be observed during the hyperventilation period, changes in pulse pressure (PP) appear to mirror changes in mean arterial pressure (MAP), and Bulk brain tissue pulsations (BTP) Amplitude shows similar trends for both left and right hemispheres. There appears to be a decrease in Bulk BTP Amplitude and variability during the recovery phase compared to the hyperventilation phase. The brackets on the x-axis indicate the 30 s time intervals used in statistical analysis for this recording. All 30 s intervals were chosen to be close to the end of each phase, while also avoiding the inclusion of artefacts.

Time series for an entire recording are shown in Figure 2, including an indication of the 30 s intervals taken forward for further analysis. For each 30 s interval, data were visually inspected and beat-to-beat values were extracted. The following features were chosen: median value for PP, MAP, HR, and EtCO_2_, and an average value for Bulk BTP Amplitude. This gave five features at each stage of the protocol (baseline, hyperventilation, and recovery) for each participant. Data were imported to Stata 12 for further statistical analysis. The distribution of Bulk BTP Amplitude features across our cohort was non-normally distributed (skewed to lower values) and is therefore summarised by a median value and IQR. All other features were normally distributed across the cohort. Comparisons between hyperventilation and baseline and recovery periods were made using a paired t-test, or non-parametric Wilcoxon signed-rank test. A Bonferroni correction was used to adjust for multiple comparisons yielding an adjusted, highly conservative, *p* = 0.004 threshold for significance.

Sensitivity analysis was carried out by removing data from the four participants that did not experience a significant change in CO_2_ levels and no effect on the results was observed, therefore data from all participants were included in the analysis.

Physiological measurements were compared between baseline and hyperventilation phases, and between hyperventilation and recovery phases, using a paired t-test to quantify the magnitude and direction of changes in EtCO_2_, PP, MAP and HR. Changes in left and right BTPs are reported as differences between median BTP amplitude values, with statistically significant changes identified using a Wilcoxon signed-rank test. Potential differences in BTP amplitude between men and women were explored using a Mann-Whitney test.

## 3. Results

**Figure 3:**
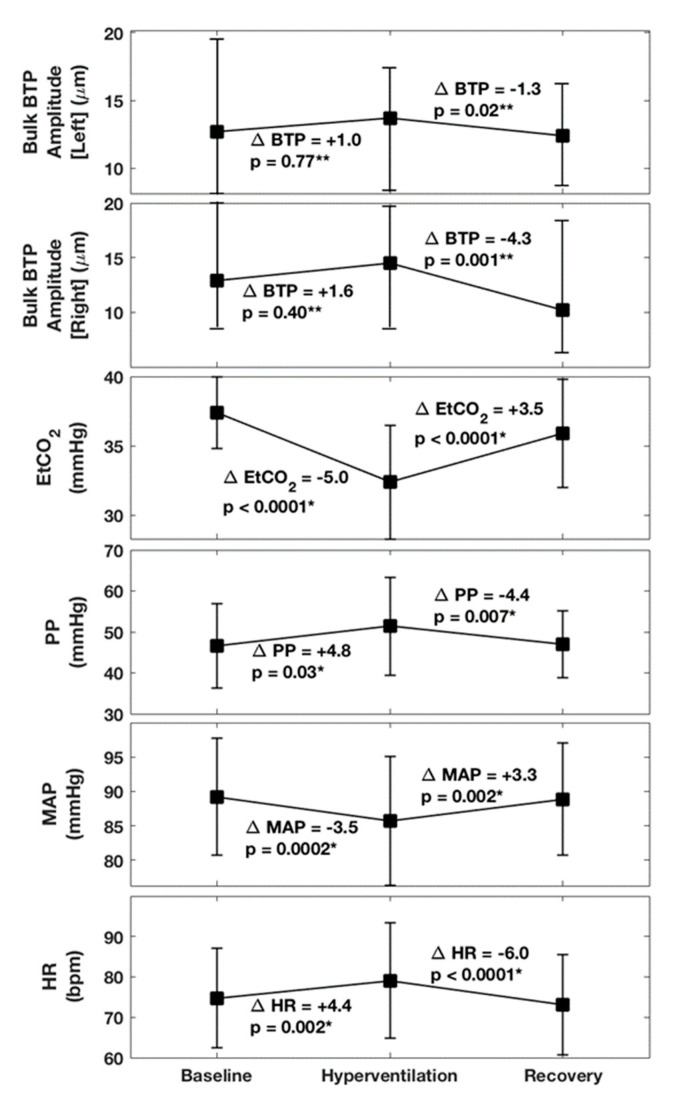
Summary of changes observed between baseline, hyperventilation, and recovery in 30 volunteers. Bulk BTP amplitude is expressed as median and IQR values, with all other variables described by their mean and standard deviation. Estimated differences between baseline and hyperventilation, and between hyperventilation and recovery, are labelled in the figure. Paired t-tests * and Wilcoxon signed-rank test ** were carried out to determine whether changes were statistically significant at a p-value of *p* = 0.05. For 95% For confidence limits, please see the values reported in the text. (Note that adjustment for multiple comparisons using a Bonferroni correction suggests an adjusted *p*-value of *p* = 0.004 as a more conservative threshold for significance.).

**Figure 4:**
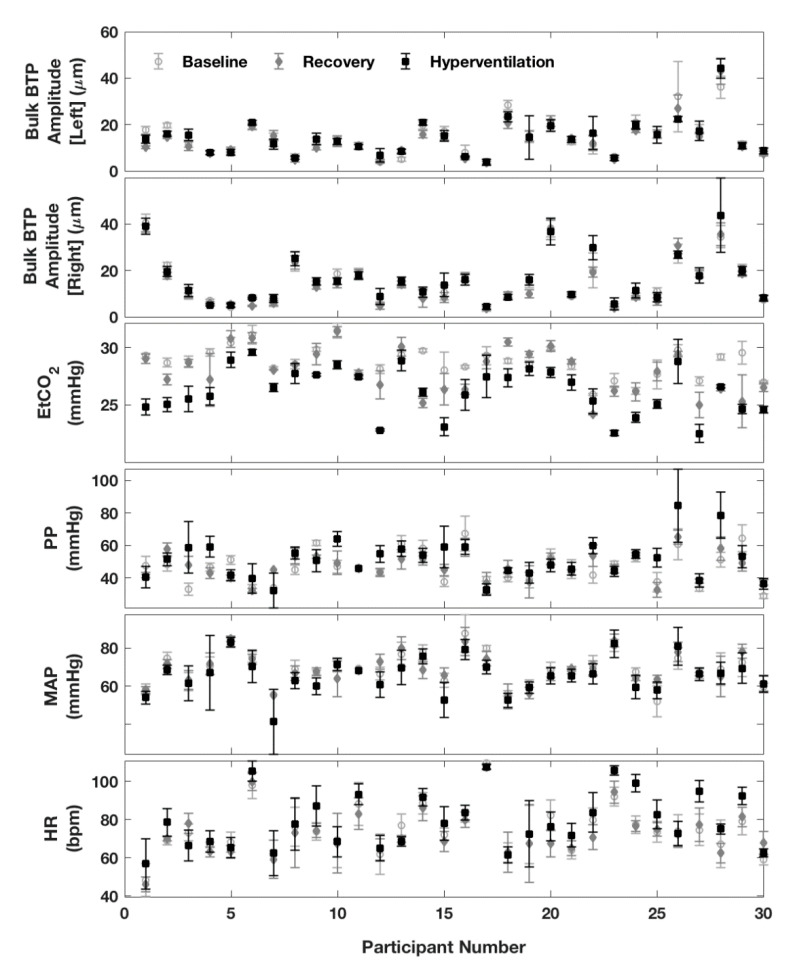
Summary of changes in variables between baseline, hyperventilation, and recovery for each participant. Graphs for Left and Right Bulk BTP Amplitude show the mean beat-to-beat value calculated over a 30 s interval, with the standard deviation (SD) indicated by error bars. Graphs for the remaining variables (EtCO_2_, PP, MAP, and HR) show the median beat-to-beat value with the IQR indicated by error bars.

Thirty healthy volunteers (12 men and 18 women) took part in this study; ages were skewed towards younger participants, median age 25 years (IQR: 24, 30), range 19 to 46 years. The hyperventilation task successfully generated a significant −5.0 mmHg (95% CI: −6.2, −3.9, *p* < 0.0001) drop in EtCO_2_, starting from a baseline mean (SD) EtCO_2_ level of 37.4 (2.6) mmHg, (see Table 1 and Figure 3 and Figure 4). In recovery, EtCO_2_ increased by +3.5 mmHg (95% CI: 2.5, 4.5 *p* < 0.0001) signalling a partial return to baseline. However, EtCO_2_ values measured during the recovery phase remained 1.6 mmHg (95% CI: 0.5, 2.6, *p* = 0.005) lower than the baseline value. Closer scrutiny of individual responses to hyperventilation revealed that responses to the breathing manoeuvre varied between participants from a maximum drop in CO_2_ levels approaching −10 mmHg to zero response (Figure 4). The cohort included four non-responders.

As expected, hyperventilation also generated significant additional responses in other physiological variables. Baseline, hyperventilation and recovery changes averaged across the entire cohort are summarised in Figure 3. Adjustment for multiple comparisons using a Bonferroni correction suggests an adjusted p-value of *p* = 0.004 as a more conservative threshold for significance. During hyperventilation PP increased by 4.8 mmHg (95% CI: 0.5, 9.0, *p* = 0.03) with an almost complete return to baseline during the recovery. Although this p-value is not below the Bonferroni corrected value of *p* = 0.004, the 95% CI suggests an increase in PP of at least 0.5 mmHg. MAP decreased by −3.5 mmHg (95% CI: −5.2, −1.8, *p* = 0.0002), also returning almost to baseline. HR increased by +4.4 bpm (95% CI: 1.7, 7.1, *p* = 0.002) and then decreases by −6.0 bpm (95% CI: −8.6, −3.4, *p* < 0.0001) in recovery (overshooting the baseline). All incidental physiological changes, except for PP, were found to remain significant after correcting for multiple hypothesis tests. Comparison of values obtained before and after hyperventilation (i.e., baseline versus recovery) confirmed no significant differences in PP, MAP, or HR, confirming the transient reversible nature of induced changes.

The brain pulsation parameter of interest, median Bulk BTP amplitude, began at baseline levels of ~13 µm, with a very slight (~1–2 µm) increase in both hemispheres during hyperventilation (Figure 3). Closer inspection of individual measurements reveals that BTP values varied widely between subjects from ~4 to 45 µm (Figure 4). Due to this variability, although trends associated with the hyperventilation were consistent across the task, a significant change in BTP was only confirmed in the recovery phase, where a statistically significant drop in BTP amplitude of −4.3 µm (−8.5%), *p* = 0.001 was identified in the right hemisphere, and a drop of −1.3 µm (−6.3%), *p* = 0.02 in the left hemisphere (Figure 3). Note that only the right-side result is robust to correction for multiple comparisons. Since comparisons were undertaken using a Wilcoxon signed-rank test, no 95% CI was available.

Comparing BTP amplitude estimates from the left and right sides suggested a slightly stronger impact of the manoeuvre on the right side. However, comparison of paired left and right median BTP values did not reveal any significant differences between paired left and right measurements during baseline, hyperventilation, or recovery. A Mann-Whitney test did not reveal any significant differences in BTP values between the men and women in this cohort.

## 4. Discussion

### 4.1. Key Findings

In this study, a brief 90 s period of mild hyperventilation was found to induce a significant reversible drop in EtCO_2_, which was accompanied by an increase in HR and PP, and a decrease in MAP. Trends in our data suggest there may be a slight increase in BTP amplitude during hyperventilation, followed by a decrease in recovery. However, the magnitude of changes in BTP associated with the hyperventilation task were small (1 to 4 µm). This is of a similar level as the participant’s beat-to-beat variability and much smaller than the variability in BTPs between participants. A statistically significant decrease in BTP between hyperventilation and recovery was noted in both hemispheres.

### 4.2. Context with Respect to Existing Literature

Comparing these results with previous findings, Kucewicz et al. [10] observed a reduction of 25-50% in brain tissue pulsatility in four participants when inducing a much larger ~20 mmHg drop in EtCO_2_ (from ~40 mmHg to ~20 mmHg) over a period of 20 min [10]. Based on our larger dataset involving 30 participants, and a more clinically relevant 5 mmHg drop in EtCO_2._ we found that BTP amplitude had potential to increase as well as decrease over the period of our experiment. Converting absolute to percentage changes, to aid direct comparison of our results with Kucewicz’s, suggested BTP changes, in individual participants can range between +71% and −31% during hyperventilation, and +77% to −42% in recovery. Monitoring over a longer hyperventilation period would be needed to confirm whether a decrease in BTP would eventually be observed during a longer hyperventilation period.

To the best of our knowledge, Kucewicz et al. [10] are the only other group who have investigated the response of BTPs to hyperventilation. Their study was limited by a small sample size (*n* = 4) and differs from the current study by invoking a much larger EtCO_2_ reduction over a longer hyperventilation period, during which other physiological processes may begin to dominate. In their study, findings for the recovery period were not reported and a different Doppler measurement system was utilised, providing a global assessment of BTP amplitude in a 2D plane rather than along a single beamline. A finding of reduced BTP amplitude during hypocapnia is expected, as hypocapnia causes vasoconstriction, with reduced arterial blood supply to the brain in systole reducing tissue expansion [2]. Our study suggests that both baseline BTP values, and responses to the CO_2_ manoeuvre differ widely between individuals. The underlying reasons for these wide variations between participants are unclear, and we are therefore currently unable to confirm the relationship between CO_2_ and BTPs.

Whilst our study improved on the previous literature by using a larger sample size of 30 participants and a wider range of additional physiological measurements, we were unable to confirm a clear relationship between BTP amplitude and hypocapnia. Overall, median BTP amplitude increased slightly (by ~1 µm) during the manoeuvre itself, with a pronounced decrease in BTP in recovery.

A much better understanding of the relationship between BTPs and other factors, such as cerebral blood flow and brain metabolic demand, will be required to explain our findings. Future research in this area should measure cerebral blood flow using conventional TCD to assess whether changes in BTPs mirror cerebral blood flow velocity changes. Cerebral autoregulation is a dynamic process, which involves various interacting parameters, making it difficult to examine the relationship between EtCO_2_ and BTPs in isolation.

Our findings suggest that a period of hypocapnia (even as short as 90 s) has the potential to cause a vasoconstrictive response that continues even beyond re-instatement of normocapnia, or indeed hypercapnia. This phenomenon was recently demonstrated in both healthy volunteers [2] and patients suffering intracerebral haemorrhage [3]. Animal studies have shown that the vasoconstrictive effect continues even after ischaemia and/or reperfusion is reversed [14]. This is thought to be driven by an increase in pH, acting directly on vascular smooth muscle via secondary messengers, which places arteries at risk of ‘vaso-paralysis’. Our findings provide evidence of hypocapnic vasoconstrictive effects leading to persistently lower BTPs.

Further work is required to confirm our findings, and to better understand how the severity and duration of hypocapnia impacts BTP amplitude. A limitation of our study is that we were unable to alter EtCO_2_ without simultaneously inducing changes in other potential confounders such as PP and HR. In a previous cross-sectional study, it was found that participants with higher pulse pressure had significantly higher BTPs; a 1% higher blood pressure was associated with a 0.8% higher Bulk BTP amplitude [15]. Visual inspection of trends within our data also suggest Bulk BTP amplitude may be positively correlated with PP and HR. It is possible that PaCO_2_ does reduce BTP amplitude over a longer hyperventilation duration, but that the impact of CO_2_ was obscured in our experiment by larger increases in BTP associated with changes in other parameters, particularly PP. A more sophisticated statistical model based on information from further experiments with other variables under controlled conditions would be needed to better understand this parameter space.

This study found that MAP decreased with hypocapnia. This was an anticipated result and agrees with previous CO_2_ studies which found a similar association [2,3,16]. Interestingly, this decrease in MAP was observed with the mild degree of hypocapnic change generated in this study. Whilst blood pressure has previously been shown to affect BTP measurements [8], it is important to further consider why changes in MAP occurred in our study without a significant effect on BTP amplitude.

Finally, this study also found an increase in PP with hypocapnia. The relationship between hypocapnia and PP is rarely explored in existing literature. However, assessment of orthostatic intolerance (OI) during hyperventilatory states suggest similar increases in pulse pressure during hypocapnia [17]. In previous research, PP changes were observed in both controls and in patients suffering OI [17]. Interestingly, hyperventilation counteracted adverse physiological changes that occur during orthostatic stress for patients suffering OI, including higher HR and lower PP [17]. The relationship between hypocapnia, PP and BTPs is clearly complex and therefore warrants further investigation.

### 4.3. Clinical Application

It has been suggested that BTP measurements may be of clinical utility in conditions such as stroke or depression [5]. However, critically unwell patients may experience changes in EtCO_2_ and PP [18]. Therefore, defining the effects of physiological changes on BTP amplitudes is an important area of research, not only to provide a better understanding of BTPs, but also to allow appropriate adjustments for clinical use of BTP measurements.

For example, a recent meta-analysis found that patients with acute stroke demonstrate mild hypocapnia (a pooled EtCO_2_ value of −1.28 mmHg compared to baseline) [19]. Applying the findings from our study on induced mild hypocapnic changes in healthy participants, we find it unlikely that the observed hypocapnia in these patients would be responsible for major BTP changes. If BTP changes are observed, these are more likely due to other cerebral haemodynamic factors, or structural tissue disruption secondary to vasogenic oedema, infarction, or haemorrhage.

### 4.4. Limitations

Whilst this study expands on previous BTP work and uses well-established hypocapnic intervention methods [2], there are some limitations which should be overcome in future work. Firstly, the population used in this study were healthy volunteers, with an average age skewed towards younger participants. As cerebral autoregulation can vary with age [20], future studies should investigate a wider age range. This would provide a clearer understanding of the relationship between CO_2_ and BTPs. More elderly patients should be recruited, as many of the proposed clinical uses for BTP measurements (including cerebrovascular disease and stroke) occur more frequently in an elderly population.

Another limitation of this study was that a small hypocapnic range was explored. Only a reduction of 5 mmHg was investigated, meaning that the expected hypocapnic range for common disease was not fully explored. Future studies could aim to explore a wider range of EtCO_2_ values, including hypocapnic changes, but also hypercapnia using inhaled CO_2_. This would allow dose-response modelling of the impact of CO_2_ on BTP measurements, whilst also allowing testing of previous work by Kucewicz et al. at the extremes of induced hypocapnia [10].

Finally, it is worth noting that a nasal cannula was used for EtCO_2_ measurements in this study. Whilst not a limitation of the study methods, care must be taken in future work in the selection of EtCO_2_ measurement techniques. Previous research has highlighted discrepancies in measured EtCO_2_ levels between measurements made using nasal cannulae vs. a face mask [1]. Both this study and the previous work of Kucewicz et al. used nasal cannulae. Finally, simultaneous measurement of cerebral blood flow using conventional TCD may be a useful addition to the monitoring in order to provide a more complete picture of cerebral haemodynamic changes and autoregulation.

As our TCTD measurements were obtained through the forehead (away from the temporal acoustic windows) we were unable to detect blood flow through the skull. This makes it difficult to exclude the possibility of BTP estimates being locally affected by the presence of major pulsating arteries, such as the anterior cerebral artery (ACA). To check the positioning of the probe in relation to large arteries, future studies should combine TCTD with MR angiography measurements showing the positions of the major arteries relative to the ultrasound beam.

## 5. Conclusions

This healthy participant study showed changes in BTP amplitude associated with recovery from mild transient hypocapnia. However, physiological responses to the manoeuvre varied considerably between participants, and since significant changes in PP, HR and MAP were also recorded, it was difficult to confidently determine the true impact and time course of CO_2_-induced BTP changes. The results of this study may assist in exploring the clinical utility of BTP measurements as they suggest that mild hypocapnic changes are unlikely to be a major cause of BTP disruption.

## Figures and Tables

**Table 1:** Hyperventilation for 90 s successfully reduced EtCO_2_ by −5.0 mmHg and generated significant transient perturbations in PP, MAP and HR. Some evidence of an impact of hyperventilation on BTPs was found when inspecting trends, and a significant drop in BTP amplitude was noted for the right and left hemispheres in the recovery phase. A summary of the results of hypothesis tests comparing hyperventilation phases with baseline and recovery is provided in Figure 3.

	Baseline	Hyperventilation	Recovery
EtCO_2_ mean (SD), mmHg	37.4 (2.6)	32.4 (4.1)	35.9 (3.9)
Left Bulk BTP Amplitude median (IQR), µm	12.7 (8.0, 19.4)	13.7 (8.4, 17.3)	12.4 (8.7, 15.9)
Right Bulk BTP Amplitude median (IQR), µm	12.9 (8.6, 20.0)	14.5 (8.5, 19.5)	10.2 (6.6, 18.2)
PP mean (SD), mmHg	46.6 (10.2)	51.4 (11.9)	47.0 (8.1)
MAP mean (SD), mmHg	89.2 (8.5)	85.7 (9.4)	88.9 (8.2)
HR mean (SD), bpm	74.7 (12.3)	79.1 (14.3)	73.1 (12.4)
